# Accessibility and implementation in UK services of an effective depression relapse prevention programme – mindfulness-based cognitive therapy (MBCT): ASPIRE study protocol

**DOI:** 10.1186/1748-5908-9-62

**Published:** 2014-05-24

**Authors:** Jo Rycroft-Malone, Rob Anderson, Rebecca S Crane, Andy Gibson, Felix Gradinger, Heledd Owen Griffiths, Stewart Mercer, Willem Kuyken

**Affiliations:** 1 School of Healthcare Sciences, Bangor University, Bangor, Gwynedd LL57 2EF, UK; 2 National Institute for Health Research (NIHR) Collaboration for Leadership in Applied Health Research and Care (CLAHRC) for the South West Peninsula, Peninsula Technology Assessment Group (PenTAG), Institute for Health Services Research, University of Exeter Medical School, Exeter EX4 4QG, UK; 3 Centre for Mindfulness Research and Practice, School of Psychology, Dean St Building, Bangor University, Bangor, Gwynedd LL57 1UT, UK; 4 Mood Disorders Centre, Psychology, University of Exeter, Exeter EX4 4QG, UK; 5 Institute of Health and Wellbeing, General Practice and Primary Care, University of Glasgow, 1 Horselethill Road, Glasgow G12 9LX, UK; 6 Mood Disorders Centre, University of Exeter, Exeter EX4 4QG, UK

**Keywords:** Mindfulness-based cognitive therapy (MBCT), Depression, Mental health, Implementation, Qualitative research, PARIHS framework, NICE guidelines, NHS

## Abstract

**Background:**

Mindfulness-based cognitive therapy (MBCT) is a cost-effective psychosocial prevention programme that helps people with recurrent depression stay well in the long term. It was singled out in the 2009 National Institute for Health and Clinical Excellence (NICE) Depression Guideline as a key priority for implementation. Despite good evidence and guideline recommendations, its roll-out and accessibility across the UK appears to be limited and inequitably distributed. The study aims to describe the current state of MBCT accessibility and implementation across the UK, develop an explanatory framework of what is hindering and facilitating its progress in different areas, and develop an Implementation Plan and related resources to promote better and more equitable availability and use of MBCT within the UK National Health Service.

**Methods/Design:**

This project is a two-phase qualitative, exploratory and explanatory research study, using an interview survey and in-depth case studies theoretically underpinned by the Promoting Action on Implementation in Health Services (PARIHS) framework. Interviews will be conducted with stakeholders involved in commissioning, managing and implementing MBCT services in each of the four UK countries, and will include areas where MBCT services are being implemented successfully and where implementation is not working well. In-depth case studies will be undertaken on a range of MBCT services to develop a detailed understanding of the barriers and facilitators to implementation. Guided by the study’s conceptual framework, data will be synthesized across Phase 1 and Phase 2 to develop a fit for purpose implementation plan.

**Discussion:**

Promoting the uptake of evidence-based treatments into routine practice and understanding what influences these processes has the potential to support the adoption and spread of nationally recommended interventions like MBCT. This study could inform a larger scale implementation trial and feed into future implementation of MBCT with other long-term conditions and associated co-morbidities. It could also inform the implementation of interventions that are acceptable and effective, but are not widely accessible or implemented.

## Background

Depression is a major public health problem that, like other chronic conditions, typically runs a relapsing and recurring course, producing substantial decrements in health and considerable human suffering [1,2]. In terms of disability-adjusted life years, the World Health Organization consistently lists depression in the top five disabling conditions [3] and in terms of years lost to disability amongst the top two, and forecasts that this will worsen over time [4]. While 23% of the total burden of disease is attributable to mental health problems, only 13% of NHS health expenditure is spent on mental health [5]. Health economic analyses of the cost of anxiety and depression in the UK suggest a cost of £17 billion or 1.5% of the UK gross domestic product [5,6]. A major factor contributing to the economic effects of depression is the reduced capacity that sufferers have to engage in the work-place.

Without effective treatment, people suffering recurrent depression have a high risk of repeated lifetime depressive episodes. The substantial health burden attributable to depression could be offset through making accessible evidence-based interventions that prevent depressive relapse among people at high risk of recurrent episodes [7]. Currently, the majority of depression is treated in primary care, and maintenance antidepressants are the mainstay approach to preventing relapse. To stay well, the recently re-named National Institute for Health and Care Excellence (NICE) recommends that people with a history of recurrent depression continue antidepressants for at least two years [8]. However, there are many drivers for the use of psychosocial interventions that provide long-term protection against relapse [9]. The majority of patients express a preference for psychosocial approaches that can help them stay well in the long-term and find that antidepressant medication can have unwanted side effects. The rates of adherence to medication regimes tend to be poor and in the perinatal period many women prefer an alternative to psychotropic medication [9].

### Mindfulness-based cognitive therapy (MBCT)

To address this need, mindfulness-based cognitive therapy was developed as a psychosocial intervention intended to teach people with a history of depression the skills to stay well in the long term [10]. Mindfulness-based cognitive therapy is a manualized psychosocial, group-based relapse prevention programme for people with a history of depression who wish to learn long-term skills for staying well [11]. It combines systematic mindfulness training with elements from cognitive-behavioural therapy. It is taught in classes of 8 to 15 people over eight weeks. Through the mindfulness course, people learn new ways of responding that are more self-compassionate, nourishing and constructive. This is especially helpful at times of potential depressive relapse, when patients learn to recognise habitual ways of thinking and behaving that tend to increase the likelihood of relapse and can choose instead to respond adaptively.

A systematic review and meta-analysis of six randomised controlled trials (N = 593) suggests mindfulness-based cognitive therapy significantly reduces the rates of depressive relapse compared with usual care or placebo controls, corresponding to a relative risk reduction of 34% (risk ratio 0.66, 95% confidence intervals 0.53 to 0.82) [12]. This is consistent with NICE’s conclusion, ‘Of the treatments specifically designed to reduce relapse group-based mindfulness-based cognitive therapy has the strongest evidence base with evidence that it is likely to be effective in people who have experienced three or more depressive episodes’ [8]. This recommendation is mirrored by the Scottish Intercollegiate Guidelines Network guideline for the non-pharmaceutical management of depression in adults [13].

There is preliminary evidence that MBCT is cost-effective compared with the current treatment of choice, maintenance antidepressants [14]. There is evidence of its acceptability to patients and referrers [15,16]. The UK Network for Mindfulness-based Teacher Training Organisations has set out good practice guidelines for training and supervision (see: http://mindfulnessteachersuk.org.uk/#welcome).

In line with the MRC Complex Interventions Framework and leading commentators [17], the next phase of work is to determine how MBCT can be implemented in ‘uncontrolled real world’ healthcare settings [18]. A search in Web of Knowledge, Science Direct and Google Scholar using the terms ‘implementation + or knowledge transfer+’, ‘Mindfulness-Based Cognitive Therapy’, ‘MBCT’, ‘mindfulness’, ‘mindfulness + knowledge transfer’ yielded only five studies with a focus on implementation processes [19-23]. Therefore, the potential to create new knowledge in this study is significant.

### Feasibility work

One of the two extant implementation studies was completed as a feasibility study for this project by two of the applicants [24]. This study asked to what extent MBCT has been implemented in the health service to date and what had facilitated implementation. It was based on: a stakeholder workshop (N = 57), a postal survey (N = 103), and an overview of four services that had either partially or fully integrated MBCT services. The results suggested that accessibility across the UK is very limited. A total of 81% of respondents reported that the implementation of MBCT had not yet begun in their organization. Where implementation had started, very few respondents reported a strategic and systematic approach to implementation. Instead, successful implementation was most frequently described as being due to ‘enthusiasts’ who had driven through change, but that these initiatives largely lacked organizational commitment or integration with other services. The authors note that the limited implementation of MBCT contributes to health inequalities and misses an opportunity to translate evidence into practice. This feasibility study was based on convenience samples and was largely descriptive. It also does not offer an explanation of why MBCT implementation to date is so patchy and inequitably distributed – hence the need for this study.

### Research aims

Even if a psychosocial intervention has compelling aims, has been shown to work, is cost-effective and is recommended by a national advisory body, its value is determined by how widely available it is in the health service. Feasibility work completed in preparation for this study indicates that NHS provision of MBCT falls well short of that envisaged in national guidance [24]. A recent British Medical Journal editorial suggests that research is needed to answer the questions, ‘What are the facilitators and barriers to implementation of NICE’s recommendations for MBCT in the UK’s health services? Can this knowledge be used to develop an Implementation Plan for introducing MBCT consistently into NHS service delivery?’ [18]. Moreover, NHS England has made ‘improving access to psychological therapies’ a priority in order to focus effort and resources on improving clinical services and health outcomes [25]. The recently launched Parity of Esteem programme (http://www.england.nhs.uk/ourwork/qual-clin-lead/pe/) has ‘a national ambition by end March 2015 to increase access so that at least 15% of those with anxiety or depression have access to a clinically proven talking therapy services, and that those services will achieve 50% recovery rates’. Similar policy pledges in other UK countries aim at improving access to psychological therapies with a specific focus on prevention, *e.g*., amongst the six high level outcomes in the Welsh Strategy ‘Together for Mental Health’, one is: ‘Access to, and the quality of preventative measures, early intervention and treatment services are improved and more people recover as a result’ [26]. There is a growing commitment amongst policy makers, commissioners, and those delivering services to ensuring that people with mental health problems receive the evidence-based treatments they need, for example as captured in the commitments of the Mental Health Strategy for Scotland 2012 to 2015 [27], or the standards of the Service Framework for Mental Health and Wellbeing in Northern Ireland from 2011 [28]. This is mirrored in patient advocacy groups calling for greater access to and choice in psychosocial treatments.

This research will describe the current state of MBCT implementation across the UK and develop an explanatory framework of what is hindering and facilitating its progress. From this framework, we will develop an Implementation Plan and related resources to promote wider access to and use of MBCT.

Specifically, we will:

1. Scope existing provision of MBCT in the health service across England, Northern Ireland, Scotland and Wales.

2. Develop an understanding of the perceived benefits and costs of embedding MBCT in mental health services.

3. Explore facilitators that have enabled services to deliver MBCT.

4. Explore barriers that have prevented MBCT being delivered in services.

5. Articulate the critical success factors for enhanced accessibility and the routine and successful use of MBCT as recommended by NICE.

6. Synthesize the evidence from these data sources, and in cooperation with stakeholders, develop an Implementation Plan and related resources that services can use to facilitate the implementation of MBCT.

## Methods/Design

**Figure 1:**
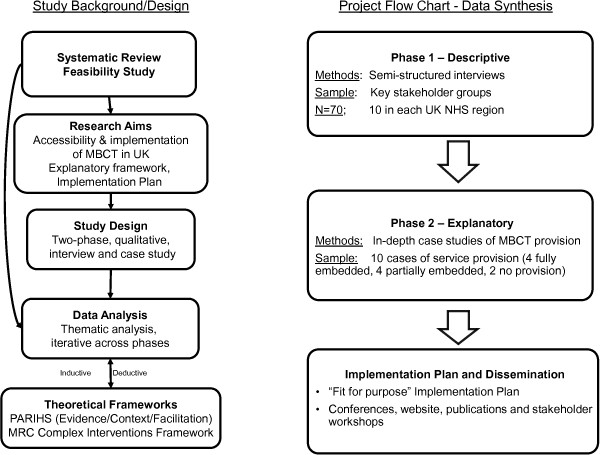
Study overview.

The planned work is a two-phase exploratory and explanatory research study, using an interview survey and in-depth case studies. An overview of this process is provided in Figure 1.

### Design and theoretical framework

We will use the Promoting Action on the Implementation of Research in Health Services (PARIHS) to underpin this study, where successful implementation is represented as a function of the interaction between evidence, context and facilitation [29,30]. PARIHS is particularly relevant to this study because it provides a conceptual map of what requires attention to ensure successful MBCT implementation, including evidence (*e.g*., NICE recommendations), context (what facilitates and inhibits evidence use - at micro [individual], meso [team], and macro [service] levels) and facilitation (what mechanisms/approaches/strategies have been helpful in enabling services to deliver MBCT).

### Approach

This is a two-phase exploratory and explanatory research study, using an interview survey and case studies [31].

### Phase 1 – interview survey

This phase will scope existing provision of MBCT, ascertain views about embedding MBCT into service delivery, including models of teacher training, facilitators, barriers, costs and benefits. The findings from this phase will give us a broad and high level perspective on if, and how MBCT is being delivered across the four countries of the UK, including the factors that have facilitated and/or hindered its implementation at the level of commissioning and service delivery. We will use telephone and face-to-face (as convenient to participants) interviews with a range of stakeholders across UK services.

### Phase 2 – case studies

In-depth case studies using exploratory and interpretive methods will be conducted. In this study, a ‘case’ is defined as an NHS Trust, Health Board or commissioned organization where NICE/SIGN recommendations would suggest there should be MBCT provision free at the point of delivery. In contrast to Phase 1, which will provide a broad and overarching perspective of MBCT service delivery in the UK, Phase 2 will provide an in-depth and contextually rich description of how MBCT becomes embedded (or not) within local service delivery. We have therefore chosen to conduct Phase 2 through mixed methods case studies. Case study is a particularly useful approach to understanding how interventions and initiatives operate within the ‘real life’ of practice and policy, and for making sense of complex individual, social and organizational phenomena where the investigator has little or no control over the practices or strategies under investigation [31]. MBCT is a complex intervention involving individuals, teams and organizations in multiple and dynamic ways, and case study methods provide an ideal approach for obtaining a rich understanding of implementation processes. For example, MBCT has a number of components that build on each other; it should sit within care pathways for common mental health problems alongside other evidence-based treatments such as medication and cognitive-behavioural therapy; it relies on a range of individuals and organizations to train and supervise MBCT therapists; it targets more than one outcome (*e.g*., relapse prevention and quality of life); and, while MBCT is manualized, it is sometimes tailored to specific contexts/populations. The team has extensive experience in conducting case study research resulting in the development of new insights, and in the development of theory [32,33].

### Sampling

This study is of relevance to commissioners, service managers, MBCT practitioners, referrers, people living with depression, and carers. Therefore, they will make up the stakeholder group that we will include in Phase 1 and 2 data collection, data synthesis, and in our engagement and dissemination strategy.

### Phase 1

Interviewees will include commissioners, managers, MBCT teachers, referrers, and people living with depression. The UK provides an opportunity for a ‘natural experiment’ in that we propose to interview stakeholders from NHS regions from across the four UK devolved administrations to provide a broad perspective on MBCT implementation within respective, different policy contexts, and operating health service environments. Gatekeepers have been identified within regions based on our knowledge of MBCT implementation through the provision of training, supervision and consultancy to NHS services. Sampling ensures the inclusion of a variety of stakeholders with criteria being developed to include different roles, and involvement in the delivery of MBCT services.

The sampling frame for interviews ensures the inclusion of relevant stakeholders from each geographical NHS region. Within each area, we will begin with a stakeholder who has knowledge of MBCT service delivery across their region, and will then seek out other stakeholders who are involved in the delivery of MBCT services, in commissioning the service, have used the service (*i.e*., people living with depression), or refer to the service to enable us to scope existing provision across the UK. Within the purposively sampled pool of eligible interviewees, we will sample at random. Our preparatory work has involved securing permission from a key stakeholder in each region. In addition to the identified stakeholder, we propose to interview up to 9 additional people in each of the following NHS regions: England North, Midlands, South and London, Wales, Scotland and Northern Ireland (*i.e*., a sample of up to 70 people). We will stop interviews within the regions when we are confident we have a comprehensive picture of service delivery in that area, and in consultation with the Project Advisory Group.

### Phase 2

We will sample ten cases to enable the differing UK service structures and contexts to be represented. A ‘case’ is defined as an NHS Trust, Health Board or commissioned organization where NICE recommendations would suggest that there should be MBCT provision free at the point of delivery. Within cases, data will be collected to include the perspectives of local commissioners, managers, MBCT teachers, referrers, practitioners and people living with depression.

Criteria for sampling include:

1. Geographic area. We will sample sites across Northern Ireland, Scotland, Wales and England.

2. Extent of MBCT being embedded in service delivery. Criteria about ‘embeddedness’ will be developed by considering the key features of ‘best practice’ in MBCT and how those should translate in to service delivery. We will include four sites where MBCT has been integrally embedded, and intend to spend up to four weeks within the site intensively collecting data. Here, it is likely that we will seek to recruit cases where key features of best practice are present: *e.g*., the organization has an explicit strategy for MBCT implementation; clinicians have been trained to teach MBCT to minimum practice levels; MBCT classes are accessible as evidenced by throughput of clients and predictable availability of provision; and referrers are informed and knowledgeable about MBCT service provision.

A further four cases will be identified and approached for recruitment where MBCT implementation has been partial. These sites are likely to be characterized by the absence of a compelling organizational strategy for implementation, MBCT teachers working in isolation, or the organization has an explicit strategy but is at an early stage in implementing it. Our understanding from contact with stakeholders in these sites is that the narrative may be more limited. Therefore, we intend to spend up to two weeks in these sites collecting data.

Finally, we will sample two sites where there is no or scarce MBCT implementation. These sites are characterized by the absence of any MBCT provision free at the point of delivery or where delivery is partially or wholly funded by charging patients (*i.e*., out-of-pocket). We intend to spend up to two weeks in these sites collecting data.

Across the ten sites we will endeavour to have a sample representative of the UK population with respect to socio-demographic profile, deprivation index, prevalence of mental health problems, urban vs. rural, and ethnic profile, which provides a theoretically transferable context.

Based on the above criteria, sites have been approached and their agreement in principle to participate secured. Permission has been secured from more sites than are needed, enabling us to choose which sites to use based on outcomes in Phase 1, the contextual analysis of each site, and following this random selection. We have also shared our data collection plans with potential sites, to assess feasibility. Potential participants have indicated that the proposed research would be acceptable and viable.

Within the sites, we will use criterion sampling to identify participants and data collection opportunities.

Criteria include:

1. Different stakeholder views about MBCT delivery locally – including from managers, people living with depression, practitioners, teachers, referrers and commissioners.

2. Level in organization – to ensure macro, meso, micro levels (as outlined above) of the organization is included.

As requested by the funder, when we have a list of potential participants, we will randomly sample potential interviewees.

### Data collection

This study will use two linked qualitative research studies.

Phase 1 will be used to scope existing services, begin to understand perceived benefits, resource implications and costs of embedding MBCT in services, and begin to explore facilitators and barriers to implementation. In line with Grol’s approach to quality improvement in healthcare [34], we will use established benchmarks of what a good MBCT service should comprise to inform the interview schedule. We will conduct semi-structured telephone or face-to-face interviews with stakeholders from geographically representative services across the UK (as described above). A semi-structured interview schedule will be developed that focuses on describing extant services, perceptions about existing provision of MBCT, ascertaining views about embedding MBCT into service delivery, including models of teacher training, facilitators, barriers, costs and benefits. The interview schedule will also ensure the opportunity for interviewees to provide additional information about service delivery not guided by the schedule. Interviews will be audio-recorded. Emerging findings from Phase 1 will be used to inform choice of case studies and develop data collection tools for Phase 2.

Phase 2 is concerned with gaining an in-depth and rich understanding of MBCT implementation in local service delivery. Therefore, data will be collected to ensure description, explanation, and will enable the articulation of critical success factors for the routine and successful implementation of a best practice MBCT service that helps people with recurrent depression stay well in the long-term [8,18].

Within each site, a number of data collection methods will be used concurrently:

### Semi-structured interviews

In each site, up to 20 interviews will be conducted either face-to-face or by telephone (at the interviewees’ convenience), and will be audio-recorded. Based on our previous case study research [35,36], we anticipate that a maximum of 20 interviews will provide both the depth and breadth of information about an issue. This number is also practical within the timeframe of the project and not too burdensome on sites.

A semi-structured interview schedule will be developed to explore how MBCT services were developed, how they are delivered, how they were/are being implemented (*e.g*., strategies and approaches), who was/is engaged in implementation, and how services are being evaluated. The schedule will also be informed by emerging findings from Phase 1, so that issues that emerged at this stage can be explored in more depth. Additionally, we want to know what impedes the introduction, development, accessibility and routine use of MBCT because this will provide valuable information for the development of an MBCT Implementation Plan. This will include exploring where barriers to access exist even where there are MBCT services. For example, our members of the public that reviewed the outline proposal highlighted difficulties in obtaining a referral as key, in several cases even where there was a service. Finally, we want to understand what audit and evaluation procedures are routinely used by primary care and MBCT services to monitor referrals, costs and outcomes.

### Non-participant observation

Non-partisan observation of relevant naturally occurring meetings and events within each site will be undertaken, such as MBCT implementation steering group, depression pathway steering group, commissioner monitoring meetings, clinical special interest/supervision groups, or relevant meetings of people living with depression. Observations will provide a supplementary source of data to the interviews by providing a view of context-related issues, including how organizations and services are responding to the challenge of implementing MBCT. As these are naturally occurring meetings and events, we cannot anticipate how many observations will be conducted.

We will use Spradley’s nine dimensions (1980) of observation to guide the focus of data collection, which include Space, Actors, Activities, Objects, Acts, Time, Events, Goals and Feelings [37]. These dimensions have been used successfully in other projects to record useful information about processes, content and interactions. Observations will be written up as field notes.

### Documentary analysis

Relevant to (a) implementation (*e.g*., plans, pathways, guidance), and (b) context of implementation (*e.g*., National policy guidelines, success stories, critical events/incidents, outputs, changes in organization), documentary analysis will be collected. These will provide information with which to further contextualize findings, provide insight into influences of implementation, and help explanation building.

### Context analysis

This will include using national databases and census data to establish the socioeconomic distribution, ethnic profile and rates of mental health problems of the population that the case study services serve. Contextual qualitative data generated from our study combined with publicly available quantitative data will be collected and reported on regional levels (NHS commissioning regions, and health boards in Wales and Scotland). This will enable us to provide a profile of the (macro) context for each case study and ensure that we have a representative set of case studies with respect to these variables. This profiling will be completed before the set of cases to be studied is finalised.

### Synthesis and development of an MBCT implementation framework and strategy

The data collected across Phases 1 and 2 will be synthesized to develop a fit for purpose implementation framework and strategy, *i.e*., an Implementation Plan. The design and content of the MBCT Implementation Plan will be developed in consultation with the Project Advisory and Patient and Public Involvement Groups and in the light of the Phase 1 and Phase 2 findings. In addition to the evidence gathered in Phase 1 and 2, the synthesis will also be informed by high quality implementation science reviews, evidence syntheses [38-40], and the emerging small scale MBCT implementation studies [20-23]. Where there are established factors known to enhance implementation, these will be incorporated into the synthesis and Implementation Plan (*e.g*., addressing structural barriers, additional resources, engaging opinion leaders, awareness building, community engagement, establishing appropriate baseline measures and intentions for evaluation).

Whilst we will not pre-empt the exact detail of its content, we envisage that the Implementation Plan will be developed and disseminated (and thereby co-owned) with key stakeholders and will have a simple set of pathways of access aiming to be intuitive and accessible to the diverse range of audiences for whom it will be useful. It will comprise at minimum a suite of resources developed from our research findings, including strategies for successful implementation, implementation approaches, training manuals, and measurement/evaluation tools. Engagement with the stakeholder groups will ensure the MBCT Implementation Plan is relevant, accessible, co-owned, and of high utility to service providers to facilitate more successful implementation of MBCT into service delivery. It will also enable the impact of implementation to be measured against meaningful benchmarks and outcomes. It may also specify which service components or implementation steps are adaptable, or may be more flexibly provided in certain contexts without any risk to overall outcomes.

### Data analysis

Data from interviews, observations and documents will be analysed using a thematic analysis approach informed by Ritchie and Spencer [41], and Yin [31]. A process of inductive and deductive analysis will be undertaken informed by Ritchie and Spencer’s approach to analysis (1994), specifically, their approach to concept identification and thematic framework development. We will use the data from the interviews as the main source of information, and look for refutational or complementary findings from observations and documents. Qualitative audio-recorded data will be transcribed in full, and managed in qualitative data processing software.

First, data will be analysed within data set (interviews, observations, documents). A number of transcripts will be coded inductively, and these codes used to develop an analysis framework. The framework will be used to code the remaining data and will be refined as new codes emerge. Second, the findings that emerged within the data set will be reviewed and mapped against the key elements of the study’s conceptual framework. This will result in the development of higher-level themes.

Consistent with comparative case study, each case will be regarded as a ‘whole study’ in which convergent evidence is sought and then considered across multiple cases [31]. As such, a pattern matching logic, based on explanation-building will be used. This strategy will allow for an iterative process of analysis across sites and will enable an explanation about MBCT implementation to emerge – what works, and what has not worked, and importantly, why. It will be imperative to ensure that data analysis reflects the variety of data sources and the potential insight that each could offer in meeting the study objectives. Analysis will first be conducted within sites and then to enable conclusions to be drawn for the study as a whole, findings will be summarized across sites.

The study’s PARIHS conceptual framework will facilitate data integration within and across phases in that it will provide a heuristic for managing the themes from the various sources of information. Use of the framework will also provide potential opportunities for theory evaluation and development. Several members of the research team will carry out the analysis process, which will include cross checking, coding and theming. Emerging themes will also be shared periodically with the whole research team, including the patient and public involvement team, as an additional check on credibility. At various stages, the stakeholder groups will provide input on the emergent analysis.

### Patient and public involvement (PPI)

When PPI is at the heart of research and service development, it promotes equity, excellence, and a sense of shared ownership [42-44]. Two recent systematic reviews reported the most beneficial impacts in the research stages of agenda setting, design and delivery, recruitment, and dissemination [45,46]. A recent study also provides evidence that higher levels of PPI in mental health research projects are associated with higher levels of recruitment to research studies [47]. With regard to implementation, another earlier review in a mental health context suggests that PPI may be crucial and effective with regard to facilitating changes in organizational culture [48]. The PPI approach in this project is premised on these values and evidence. Moreover, it is informed by INVOLVE and Mental Health Research Network best practice guidelines [49-51]. We use a model of PPI that emphasizes the key dimensions of engagement with public concerns, strength of the PPI voice, and appropriate modes of engagement in different elements of the research [52].

In developing this proposal, people living with depression who have or have not participated in MBCT in the NHS were consulted. The results of this consultation informed the current plans and methodology of the project, specifically ensuring that our case study sampling is representative and looking at barriers to accessing services even where services exist.

The PPI group is comprised of four people with a history of recurrent depression, all of whom have accessed MBCT. We have ensured that this group includes at least two persons who provide critical distance on MBCT and can act as ‘critical friends’ to the project.

The PPI group, facilitated by one of the co-applicants (AG), will meet at least four times across the life of the project. Group members will also participate in the Project Advisory Group and contribute at various key phases to the study protocol, materials and outputs. All materials will be made available to the group in an accessible format before meetings. The study methodology, by definition, involves consultation with people living with depression through the Phase 1 survey interviews and Phase 2 case studies.

At an initial set up meeting, the PPI group set out terms of reference, clarified roles, and identified any support needs of group members. If PPI group members wish to attend with a supporter, they are welcome to do so. In addition, contingency plans should team members require psychological support during the project were discussed. The PenCLAHRC PPI team has developed a wide range of resources to facilitate the process of PPI (see http://clahrc-peninsula.nihr.ac.uk/penpig-resources.php).

Members of the PPI group attended an early Project Management Group meeting. They contributed to articulating values and ways of working within the research team to optimize team working, clarity, mutual trust and respect [53,54]. They also contributed to shaping the protocol, co-writing the Study Information Sheet and contributing to the Phase 1 study materials. Midway through the project, the PPI group will contribute to the analysis of the Phase 1 results and the development of the Phase 2 materials. In the last six months of the project, PPI members will be involved actively in the data analysis from Phase 2, data synthesis and preparation of the Implementation Plan and its associated resources. In the last three months of the project, we will invite members of the PPI group and other people with lived experience of depression to co-facilitate the dissemination workshops across the UK.

### Current status of project

The project is funded by the National Institute of Health Research (NIHR) Health Services and Delivery Research Programme (HS&DR - 12/64/187), managed by The NIHR Evaluation, Trials and Studies Coordinating Centre (NETSCC) in Southampton. Details of the grant can be found here: http://www.nets.nihr.ac.uk/projects/hsdr/1264187?src=hsdr1264187. At the time of manuscript submission, we have secured access to all the required interview survey and case study sites for the proposed research. We have found that individuals and sites have been highly motivated to engage. The proposal for the study was reviewed at three stages by external reviewers and by the funder’s commissioning panel prior to them recommending funding.

Approval to undertake the study has been granted by Cornwall and Plymouth Research Ethics Committee. Approval was granted on 22.08.13. (REC Ref No. 13/SW/0226). At the time of submission, we were in the process of securing approval by the various NHS Research and Development (R and D) departments via the Integrated Research Application System (IRAS) using the Coordinated System for gaining NHS Permission (CSP Ref: 134133 – ASPIRE). The study is also fully network supported by the National Institute for Health Research (NIHR), through the Mental Health Research Network (MHRN) and is registered on the NIHR clinical research portfolio. It is also supported by the Scottish Mental Health Research Network (SMHRN), and the Mental Health Research Network Cymru (MHRNC).

## Discussion

This study is concerned with producing rigorous and relevant evidence on the quality, access and organization of health services through describing the current state of MBCT implementation across the UK and developing an explanatory framework of the factors that are impacting progress towards implementing NICE guidance on MBCT. In collaboration with relevant stakeholders, it will use this research-based evidence to develop an MBCT Implementation Plan that addresses a major public health problem: depression. The Implementation Plan will comprise a suite of resources and will be developed to facilitate a tailored and flexible approach for use by GPs, service managers and clinicians. The resources will also be available in plain language so that they are accessible to the general public. In developing the implementation plan, we will aspire to achieving effects of co-ownership such as access to key channels for communication, further training and support in order to ensure greater reach of the outputs and maximize its impact beyond the life of the project itself.

This work will be of direct benefit to NHS services in providing a resource to support the implementation of a key priority identified in the 2009 NICE and 2010 SIGN depression guideline. The research will contribute to the knowledge of current practice which may feed into guideline reviews or the development of NICE quality standards. It will have the potential both to develop into a larger scale implementation trial and to inform future work on MBCT service design and planning for people with other long-term conditions [10]. Finally, the study will add to a growing field [38,55,56] that provides a framework and specific strategies for bridging the translational gap from effectiveness evidence to wider real-world implementation. As there is still much to learn about implementation within and across contexts, and in different types of services/clinical issues, this study will also extend our knowledge about implementation theory and practice.

## Competing interests

WK & RC Co-Direct the Mindfulness Network Community Interest Company and SM is a Trustee of Mindfulness Scotland. RC, WK & SM are involved in training mindfulness teachers. The authors declare no other competing interests.

## Authors’ contributions

RC, AG, WK, JRM & SM were responsible for the original proposal and secured funding for the project. WK and JRM are Joint Chief Investigators, are responsible for the project and drafted the original protocol. RC and SM are Co-investigators, FG and HOG are research staff and RA is a collaborator on the project. All the authors were involved in drafting, revising and approving the final manuscript. The study Chief Investigators are listed as first and last author and all other authors are listed alphabetically.
